# On the Presentation of Dead Children

**Published:** 1856-05

**Authors:** 


*On the Presentation of Dead Children.*—In the Edinburgh Obstetrical Society, Dr. Matthews Duncan stated that he had recently been engaged in making experiments with a view to find out the cause ot the frequent malpresentation of dead children. This fact of frequent malposition and malpresentation of dead children, had been too hastily assumed as a proof that the ordinary and favorable head-presentation was a vital action. The malposition of dead infants was thus held to demonstrate that the functions of the living child led to the frequent presentation of the head. The facts which Dr. Duncan would adduce showed that gravitation had much more to do in causing presentations than some modern writers were inclined to admit; for his experiments proved, that the healthy non-putrid child floated in a fluid of its own specific gravity, obliquely, and with the head lowest, while a fœtus which had been some time dead, generally floated in an exactly opposite position, or nearly so—namely, obliquely, with its head highest. The former position of healthy infants, was just the position it had in the womb of a healthy woman in the erect or recumbent position on the back. The latter position of dead infants, was the same reversed—that is, with the head highest instead of lowest. Of course, there were many other circumstances to be regarded in judging of the effect of this change in the specific gravity besides the change itself—for example, the period of pregnancy, and the quantity of liquor amnii, and the mobility of the child; also, the shape and position of the uterus.

Dr. Duncan then adduced a series of observations on the floating of children in a putrid or not putrid state, in a solution of salt of their own specific gravity, and the positions they took up in such solution. As to the cause of the great change indicated in these experiments, he could say little. It was known that one of the first alterations taking place in a recently dead child, was the dissolution of the brain in a fluid mass. This was probably what produced the diminution in its specific gravity, and consequent buoying up in the fluid in his experiments, or in the uterus, when the change takes place.

[*Edinburgh Med. Journal and Examiner.*

